# Antibiotic sensitivity pattern of *Staphylococcus aureus* from clinical isolates in a tertiary health institution in Kano, Northwestern Nigeria

**DOI:** 10.4314/pamj.v8i1.71050

**Published:** 2011-01-26

**Authors:** Emmanuel Onwubiko Nwankwo, Magaji Sadiq Nasiru

**Affiliations:** 1 Department of Medical Microbiology and Parasitology, Faculty of Medicine, Bayero University; 2 Department of Medical Microbiology, Aminu Kano Teaching Hospital, Kano

**Keywords:** Staphylococcus aureus, antibiotic sensitivity, Nigeria, Kano

## Abstract

**Background:**

The importance of *Staphylococcus aureus* as a persistent nosocomial and community acquired pathogen has become a global health concern. It has a remarkable capability of evolving different mechanisms of resistance to most antimicrobial agents. The aim of the present study is to establish the incidence of *S. aureus* in clinical specimens and its antibiotic sensitivity pattern to various antibiotics in this locality.

**Methods:**

One hundred and fifty consecutive isolates of *S. aureus* obtained from various clinical specimens between January and December 2009 sent to the Medical Microbiology Laboratory Department of Aminu Kano Teaching Hospital (AKTH) were confirmed by standard bacteriological procedures. Antibiotic sensitivity pattern was carried out by disc diffusion method.

**Results:**

The age group with the highest number of isolates was (0-10)yrs while wound infection had the highest frequency of *S. aureus* isolates (30.7%) in the study. Males (62.0%) were more infected than females (38.0%). The sensitivity pattern of *S. aureus* to the following antibiotics; Gentamicin, Amoxycillin/clavulanate, Streptomycin, Cloxacillin, Erythromycin, Chloramphenicol, Cotrimoxazole, Tetracycline, Penicillin, Ciprofloxacin, Ofloxacin, Levofloxacin, Ceftriaxone, Amoxycillin and vancomycin were 92.4%, 63.0%, 44.2%, 35.8%, 52.4%, 61.9%, 15.5%, 31.2%, 7.1%, 78.9%, 76.6%, 100%, 71.4%, 30.7% and 100% respectively. Methicillin resistant isolates were sensitive to Levofloxacin 93.7% and Ofloxacin 68.7%.

**Conclusion:**

The results of the present study show that the fluoroquinolones are effective in the management of *Staphylococcus aureus* infections including methicillin resistant strains in this environment.

## Background

*Staphylococcus aureus* is a gram-positive cocci, catalase and coagulase positive bacterium. *Staphylococcus aureus* cause disease through the production of toxin or through direct invasion and destruction of tissue. Infections caused by S. aureus remain a significant cause of mortality and morbidity in tropical countries [1]. The principal site of staphylococcal colonization is the anterior nares. It has been observed that if repeated cultures are performed, up to 80% of adults are found to harbor S. aureus in the nose at one time or the other. However, in most persons, the carrier state is transient, but 20 to 40% of adults remain colonized for months or even years [2]. Increased nasal colonization rates have been noted in insulin dependent diabetes [3], individuals on haemodialysis [4], those on ambulatory peritoneal dialysis [5], intravenous drug users [6] and patients receiving routine allergy injections [7]. It has also been suggested that patients with symptomatic human immunodeficiency virus infection have an increased colonization risks [8].

Staphylococci have a record of developing resistance quickly and successfully to antibiotics. This defensive response is a consequence of the acquisition and transfer of antibiotic resistance plasmids and the possession of intrinsic resistance mechanisms [9]. The importance of *Staphylococcus aureus* as a persistent nosocomial and community acquired pathogen has become a global health concern.It has a remarkable capability of evolving different mechanisms of resistance to most antimicrobial agents [10].

The emergence of antibiotic resistant bacteria constitutes a major problem in antibiotic therapy. Tihis could be attributed to unrestricted use of antibiotics in a particular environment

Classically, Methicillin resistant *Staphylococcus aureus* (MRSA) has been a nosocomial problem associated with long hospital stays, numerous or prolonged antibiotic courses, the presence of invasive devices and proximity to an already infected or colonised patient [11]. Mechanisms of resistance to β-lactam antibiotics and the fluoroquinolones have been documented [9]. The aim of the present study is to establish the incidence of S. aureus in clinical specimens and its antibiotic sensitivity pattern to various antibiotics in this locality

## Methods

One hundred and fifty consecutive isolates of *Staphylococcus aureus* obtained from various clinical specimens such as wound swab, blood culture, umbilical cord swab, urine, eye swab, ear swab, abscess, catheter tips, throat swab, pleural aspirate and skin swab between January and December 2009 and where processed at the Medical Microbiology Department of Aminu Kano Teaching Hospital (AKTH). Chi-square test was used for statistical analysis (EPI info V3.5.)

Specimens were cultured on Blood agar and manitol salt agar plates and incubated at 37oC for 24hrs. Characteristic *Staphylococcus aureus* colonies were identified by gram stain, catalase and coagulase testing according to standard bacteriological procedures [12]. A suspension of each confirmed *Staphylococcus aureus* isolates was prepared in peptone water to match 0.5 Mcfarland turbidity standards. S. aureus ATCC 25923 was used as control strain.

All confirmed S. aureus isolates were screened for methicillin resistance by inoculation of Mueller Hinton agar supplemented with 4% NaCl. The 5µg methicillin discs (oxoid, USA) were aseptically placed on the surface of the inoculated plates and incubated aerobically at 35oC for 18-24hrs. The isolates were similarly inoculated onto the surfaces of plain Mueller-Hinton agar plates and Gentamicin (10µg), Amoxycillin/clavulanate (30µg), Streptomycin (30µg), Cloxacillin (5µg), Erythromycin (15µg), Chloramphenicol (30µg), Cotrimoxazole (25µg), Tetracycline (30µg), Penicillin (10iu), Ciprofloxacin (5µg), Ofloxacin (5µg), Levofloxacin (5µg), Ceftriaxone (30µg) and Amoxycillin (30µg) discs were placed and incubated at 37oC for 24hrs.

The Zones of inhibition were measured and compared with national committee for clinical laboratory standards (NCCLS) guidelines [13]. The isolates that were resistant to methicillin (<17mm) were termed Methicillin resistant *Staphylococcus aureus* (MRSA) while those with zone of inhibition as (≥17mm) were termed susceptible. Antibiotic susceptibility tests were carried out by disc diffusion method [14]. Due to the non-availability of antibiotic discs at certain periods during the study, not all antibiotics were tested in equal numbers.

## Results

table 1 shows the age and sex distribution of patients with *Staphylococcus aureus* infection in Kano. Males (62.0%) had higher infection rate than females (38.0%). The highest frequency of isolates of *Staphylococcus aureus* occurred in the age group (0-10)yrs while the least was in the (51-60)yrs group. The difference was statistically significant (χ^2^=170.18 df=6 P<0.0001).

table 2 shows the different specimens from which *Staphylococcus aureus* was isolated. The highest number of isolates was from wound infections 46(30.7%) followed by Ear swab 32(21.3%). The least were from pleural aspirate and skin swab 1(0.07%) each.

table 3 shows the antibiotic sensitivity and resistance pattern of *Staphylococcus aureus* to various antibiotics. The highest frequency of sensitivity was observed with Levofloxacin 150(100%) followed by Ciprofloxacin 90(78.9%). The least was observed with Penicillin 6(7.1%). The number of strains tested was not equal for each antibiotic (Χ^2^=373.3 df=12 P<0.0001).

table 4 shows the antibiotic sensitivity pattern of Methicillin resistant *Staphylococcus aureus* (MRSA). Ciprofloxacin had 56.3% sensitive while Levofloxacin had 93.7% and Ofloxacin had 68.7% sensitive.

All isolates were sensitive to Vancomycin. All isolates were obtained from consecutive samples and were single strains from each patient. Sixteen isolates in all were obtained from 16 patients

## Discussion

*Staphylococcus aureus* is a very common cause of infection in hospitals and is most liable to infect new born babies, surgical patients, old and malnourished persons and patients with diabetes and other chronic diseases [15]. The highest frequency of isolates of *Staphylococcus aureus* (47.3%) in the present study was observed in the (0-10)yrs age group in which neonates and infants were included. It is believed that their immunity is not properly developed at this stage to cope with bacterial infections hence they are vulnerable and easily infected especially when hospitalized. The older children have also been observed to be more active than adults during their interaction with their playmates and while playing for hours, come in contact with various objects. In this process, they become a target to ubiquitous bacteria such as S. aureus.

However, it is not clearly understood why males were more infected than females in the present study. *Staphylococcus aureus* was found to be a frequent cause of burns and wound sepsis [16]. A study [17] at Ilorin, Nigeria reported wound infections of 38% as the highest frequency of S. aureus isolates. This agrees with the result in the present study where S. aureus also had the highest isolate of 30.7% followed by ear swab in otitis media (21.3%). *Staphylococcus aureus* develops resistance very quickly and successfully to different antimicrobials over a period of time. The highest frequency of S. aureus occurred with susceptibility to antimicrobial agent Levofloxacin (100%) followed by Ciprofloxacin (78.9%) while the least was Penicillin (7.1%).

Some years ago, Cloxacillin was highly recommended in staphylococcal infection in view of excellent in vitro sensitivity results. This could be seen from the reports at Ilorin, Nigeria [17] with 78% sensitivity and at Owerri, Nigeria [18] with 85.4% sensitivity. However, these results are at variance with the current trend in S. aureus susceptible to Cloxacillin as could be seen from the results of the present study and the reports of some other researchers [19-20] who all observed very low sensitivity percentage to Cloxacillin. This became more common with the advent of Methicillin resistance as they belong to the same class of antibiotics. The low percentage sensitivity of S. aureus observed in the present study against the following drugs; Tetracycline, Chloramphenicol, Penicillin and Cotrimoxazole was in agreement with the reports published by some workers [17-20] from Nigeria and another researcher [21] in Eritrea.

The high level resistance could be associated with earlier exposure of these drugs to isolates which may have enhanced development of resistance. There is high level antibiotic abuse in this environment arising from self-medication which is often associated with inadequate dosage and failure to comply to treatment [22], and availability of antibiotics to consumers across the counters with or without prescription [23].

In the present study, *Staphylococcus aureus* susceptibility to Ofloxacin was 76.6%. This is consistent with the reports from Abakiliki [19] and Owerri [18] all in Nigeria. *Staphylococcus aureus* sensitivity to Gentamicin in the present study was 73.4%. This compares favorably with reports published by some researchers [23,17]. Methicillin resistant *Staphylococcus aureus* (MRSA) has emerged as a serious public health problem of global concern. Screening for methicillin resistant isolates in this study showed a prevalence rate of 10.7%. This was however lower than the studies conducted in other areas in Nigeria such as Ilorin [25] 34.7% and Jos [26] 43.0%. In contrast, the prevalence of MRSA was found to be low in France (6%), Ireland (5%) and United Kingdom (2%) [27]. However, a high prevalence of 83% MRSA was reported from Pakistan [28]. This confirms the high regional variations in the findings from different countries and cities.

It had been observed that the indiscriminate use of antibiotics without prescriptions in the developing countries such as Nigeria where there are no regulatory policies in this respect has rendered the commonly used antibiotics completely ineffective in the treatment of *Staphylococcus aureus* infections [22]. It was encouraging to note that Vancomycin resistance was not observed among the isolates. However out of fifteen antibiotics that were tested against MRSA isolates, 10(66.7%) did not show any sensitive isolate.

## Conclusion

This study highlights the need for continuous surveillance of antibiotic sensitivity pattern of *Staphylococcus aureus* and Methicillin resistant *Staphylococcus aureus* (MRSA) with a view to selecting appropriate therapy.

The results obtained show that Vancomycin, Levofloxacin and Ofloxacin are recommended as first line antibiotics in the management of *Staphylococcus aureus* infections including methicillin resistant strains in this environment.

## Acknowledgments

We thank members of staff of the Department of Microbiology, Aminu Kano Teaching Hospital (AKTH), Kano, Nigeria for their cooperation and assistance during the study.

## Competing interests

The authors declare that they have no competing interests.

## Authors’ contributions

Both authors contributed equally in the collection, analysis and processing of samples as well as preparation of manuscript for publication.

## Figures and Tables

**Table 1:** Age and sex distribution of patients with *Staphylococcus aureus* infection in a tertiary institution in Kano, Nigeria

**Age group (years)**	**No. positive (%)**		
	**Males**	**Females**	**Total no. (%)**
<11	34(47.9)	37(52.2)	71(47.3)
11-20	12(63.2)	7(36.8)	19(12.7)
21-30	17(70.8)	7(29.2)	24(16.0)
31-40	11(100)	0(0)	11(7.3)
41-50	8(88.9)	1(11.1)	9(6.0)
51-60	3(75.0)	1(25.0)	4(2.6)
>60	8(66.7)	4(33.3)	12(8.0)
**Total**	**93(62%)**	**57(38%)**	**150**

**Table 2:** Distribution of *Staphylococcus aureus* in clinical isolates in a tertiary institution in Kano, Nigeria

	**Number**	**Percentage**
Wound swab (Wound infections)	46	30.7
Blood Cultures	28	18.7
Ear swab (Otitis media)	32	21.3
Eye swab(conjunctivitis)	8	5.3
Abscess	7	4.7
Urine	10	6.7
Umbilical cord swab	10	6.7
Catheter tips	4	2.7
Throat Swab	3	2.0
Pleural aspirate	1	1.0
Skin swab	1	1.0
**Total**	**150**	**100**

**Table 3:** Antibiotic sensitivity and resistance pattern of *Staphylococcus aureus* from clinical isolates in a tertiary institution in Kano, Nigeria

**Type of Antibiotic**	**No. tested**	**No. sensitive**	**% sensitive**	**% resistant**
Gentamicin	79	73	92.4	7.6
Amoxycillin/clavullanate	81	51	63.0	37.0
Streptomycin	129	57	44.2	55.8
Cloxacillin	134	48	35.8	64.2
Erythromycin	84	44	52.4	47.6
Chloramphenicol	142	88	61.9	38.1
Cotrimoxazole	84	13	15.5	84.5
Tetracycline	128	40	31.2	68.8
Penicillin	84	6	7.1	92.9
Ciprofloxacin	114	90	78.9	31.1
Ofloxacin	150	115	76.6	23.4
Levofloxacin	150	150	100	0
Ceftriaxone	140	100	71.4	28.6
Amoxycillin	130	40	30.7	69.3

**Table 4:** Antibiotic sensitivity pattern of Methicillin Resistant isolates in a tertiary institution in Kano, Nigeria

**Types of antibiotic**	**Number tested =16**	
	**No. sensitive (%)**	**No. Resistant (%)**
Gentamicin	0(0)	16(100)
Amoxycillin/clavulanate	0(0)	16(100)
Streptomycin	0(0)	16(100)
Cloxacillin	0(0)	16(100)
Erythromycin	0(0)	16(100)
Chloramphenicol	0(0)	16(100)
Cotrimoxazole	0(0)	16(100)
Tetracycline	0(0)	16(100)
Penicillin	0(0)	16(100)
Ciprofloxacin	9(56.3)	7(43.7)
Ofloxacin	11(68.7)	5(31.3)
Levofloxacin	15(93.7)	1(6.3)
Ceftriaxone	7(43.7)	9(56.3)
Amoxicillin	0(0)	16(100)
Vancomycin	16(100)	0(0)
